# Prevalence of nodal metastases in the individual lymph node stations for different T-stages in gastric cancer: a systematic review

**DOI:** 10.1007/s13304-022-01347-w

**Published:** 2022-08-13

**Authors:** M. H. S. de Jong, S. S. Gisbertz, M. I. van Berge Henegouwen, W. A. Draaisma

**Affiliations:** 1 Department of Surgery, Amsterdam UMC Location University of Amsterdam, Amsterdam, The Netherlands; 2 Department of Surgery, Jeroen Bosch Hospital (JBZ), ‘s-Hertogenbosch, The Netherlands; 3 Cancer Treatment and Quality of Life, Cancer Center Amsterdam, Amsterdam, The Netherlands

**Keywords:** Stomach neoplasms, Lymph node excision, Neoplasm staging, Gastrectomy, Adenocarcinoma

## Abstract

**Background:**

Gastrectomy with lymph node dissection is the cornerstone of curative treatment of gastric cancer. Extent of lymphadenectomy may differ depending on T-stage, as the rate of lymph node metastases may differ. The objective of this systematic review is to investigate and compare the prevalence of nodal metastases in the individual lymph node stations between different T-stages.

**Methods:**

Data reporting and structure of this systematic review follows the PRISMA checklist. The Medline and PubMed databases were systematically searched. The search included the following Mesh terms: "Stomach Neoplasms", "Lymphatic Metastasis" and "Lymph Node Excision". The primary outcome was the highest prevalence of nodal metastases per T-stage.

**Results:**

The initial search resulted in 175 eligible articles. Five articles met the inclusion criteria and were accordingly analyzed. Concerning the lymph node stations 1 to 7, the lymph nodes along the lesser gastric curvature (station 3) show the highest metastases rate (T1: 5.5%, T2: 21.9%, T3: 41.9%, T4: 71.0%). Concerning the lymph node stations 8 to 20, the lymph nodes around the common hepatic artery (station 8) show the highest metastases rate (T1: 0.8%, T2: 7.9%, T3: 14.0%, T4: 28.2%).

**Conclusion:**

An overall low prevalence of nodal metastases in the individual lymph node stations in early, T1 gastric carcinomas and an overall high prevalence in more advanced, T3 and T4 gastric carcinomas endorse a more tailored approach based on the different gastric T-stages. In addition, a less extensive lymphadenectomy seems justified in early T1 carcinoma.

**Synopsis:**

This systematic review provides an overview of the prevalence of nodal metastases for the individual lymph node stations between different T-stages, showing an overall low prevalence in early, T1 gastric carcinomas and an overall high prevalence in the more advanced, T3 and T4 gastric carcinomas.

## Background

The incidence of GC (gastric cancer) has decreased worldwide. However, it is still the fifth most common cancer overall and the third most common cause of cancer mortality [1]. The difference in the tumor biology, tumor stage at presentation, location and differences in treatment are presumed to be reasons for the difference in survival between patients [2]. The multimodal treatment of GC includes the combination of variable disease-based therapeutic components, including different extents of LND (lymphadenectomy). The recurrence rates after gastric surgery remain high (ranging from 20% to 50%) and LN (lymph node) involvement ought to have the strongest influence on recurrence and prognosis of GC [3–5]. For this reason, an accurate LND represents an essential component of GC surgical treatment.

The Japanese Gastric Cancer Association classification (JGCA) system for GC requires the resection of 20 LN stations plus the 110, 111, and 112 groups. LN stations 1–12 and station 14v are regarded as locoregional gastric lymph nodes, other node stations are, generally, considered as distant metastases. For esophageal invading tumors, station 19, 20, 110 and 111 are also classified as locoregional. In a total gastrectomy, a less extensive D1 LND consists of resection of the peri-gastric LNs; station 1 to 6, and LN station 7; a second-tier LN station. A D2 LND consists of resection of the D1 LN stations and the stations 8a, 9, 11p, 11d and 12a. In a distal gastrectomy a D1 LND consists of resection of the LN stations 1, 3, 4sb, 4d, 5, 6 and 7. In a distal gastrectomy a D2 LND includes the D1 stations and stations: 8a, 9, 11p and 12a [6]. For cancer of the upper stomach invading the greater curvature, dissection of station 10 is considered a D2 + LND following the 5^th^ edition of the JGCA guidelines. In previous editions of the JGCA guidelines this was defined as a D2 LND.

A more extensive LND ought to result in lower rates of loco regional recurrence and improved overall survival [7–9]. However, a more extensive D2 LND is accompanied with higher morbidity rates (43%-46% versus 25%-28%) and mortality rates (10%-13% versus 4%-6.5%) in comparison with a less extensive D1 LND [9, 10]. For selected early gastric tumors, endoscopic resection is feasible.

The Japanese Gastrointestinal Endoscopy Society has established criteria for endoscopic resection for early GC. Tumors confined to the mucosa (T1a), well-differentiated, non-ulcerated and ≤ 2 cm can be curatively treated by endoscopic resection, as the chance of LN metastases is negligible. Expanded criteria were defined for: (1) well-differentiated mucosal cancer without ulceration, greater than 2 cm in diameter; (2) well-differentiated mucosal cancer with ulceration, up to 3 cm in diameter; (3) undifferentiated mucosal cancer without ulceration, up to 2 cm in diameter and (4) differentiated submucosal cancer (SM1, < 500 μm), up to 3 cm in diameter. Tumors that meet the expanded criteria for endoscopic resection carry the risk of LNM. This risk should be balanced with the risk of surgery [11, 12].

The overall prevalence of LN metastases in T1 tumors is 8%–31%, while the overall prevalence of LN metastases in T2-T4 tumors is considerably higher; 45%–90% [13–15]. This difference in metastases rates suggest the necessity for a more tailored approach based on the gastric T-stage. The Japanese Guidelines advocate a D1 LND for T1a tumors and well-differentiated, < 1.5 cm T1b tumors that do not meet the criteria for endoscopic resection. While a D1 LND is accompanied with less morbidity and mortality, the question remains whether a D1 LND is adequate for early, T1-stage gastric carcinomas.

The objective of this systematic review is to investigate and compare the prevalence of nodal metastases in the individual LN stations between different T-stages.

## Methods

### Design

Data reporting and structure of this systematic review follows the PRISMA checklist [16].

### Literature search

The Medline/Pubmed database was thoroughly searched by two independent observers on June 1st 2022. If consensus could not be reached a third observer was consulted. The search included the following Mesh terms: “Stomach Neoplasms”, “Lymphatic Metastasis” and “Lymph Node Excision”. Besides Mesh terms the search included synonyms for these terms through appending “[tiab] words”. In Table 1 the search strategy is outlined in detail.

**Table 1:** Search

Literature search PUBMED	hits
("Stomach Neoplasms"[Mesh] OR gastric cancer[tiab] OR stomach carcinoma*[tiab]) AND ("Lymphatic Metastasis"[Mesh] OR Lymphatic Metastas*[tiab]) AND (pattern*[tiab] OR distribution*[tiab]) AND ("Lymph Node Excision"[Mesh] OR Lymph Node Excision*[tiab] OR Lymph Node dissection*[tiab] OR lymphadenectom*[tiab]) AND english[Language]	175

### Study selection and inclusion criteria

Studies reporting the prevalence of nodal metastases for the individual LN stations per T-stage, were included. Initially, articles were screened on eligibility based on title or abstract. Then the full-text article was retrieved and selected for inclusion if they met the following criteria: (1) randomized controlled trials, cohort studies, (2) patients who underwent gastric surgery including a LND, (3) patients with operable GC (4) studies reporting the prevalence of nodal metastases for the individual LN stations separately for the different T-stage. Systematic reviews, narrative reviews and case series and studies which do not distinguish between gastric and esophageal tumors were excluded. If the two observers had divergent ideas on any data, a third observer was asked to constitute and reach consensus.

### Outcome

The primary outcome was the highest prevalence of nodal metastases per T-stage, defined as the highest number of patients with pathological positive LNs in an individual LN station separately reported for the different gastric T-stages. The secondary outcome was the prevalence of nodal metastases in every individual LN station between different T-stages, defined as the number of patients with pathological positive LNs per LN station separately reported for the different gastric T-stages. The T-stage classification following the 7^th^ edition of the AJCC Cancer Staging Manual was used [17]. As the included articles were performed between 1989 and 2014, the 7th rather than the 8th edition of the AJCC Cancer Staging Manual was used. The classification of the nodal stations was used following the Japanese classification of gastric carcinoma [6, 18].

### Quality assessment

Two researchers (M.J. and W.D.) independently assessed the methodological quality of the included studies with the use of the CASP-checklist [Critical Appraisal Skills Program (2017)]. If consensus could not be reached a third observer (M.B.H) was consulted. This checklist contains questions regarding the methodological quality of the studies. Points were given dependent on the various answers. The articles were scored on their quality, ranged from 0 (low quality) to 12 (high quality). Articles scoring between 0 and 5 points were defined as “low quality”, between 6 and 8 points as “moderate quality” and between 9 and 12 points as “high quality”.

### Data extraction

The following information about the study design characteristics was extracted: first author, year of publication, journal of publication and study design. The following data about the patient characteristics and the surgery was extracted: number of patients, number of patients with positive LN metastases, tumor location, type of LND, what T-stage the study addressed and the outcome as mentioned above.

## Results

### Study selection

**Fig. 1:**
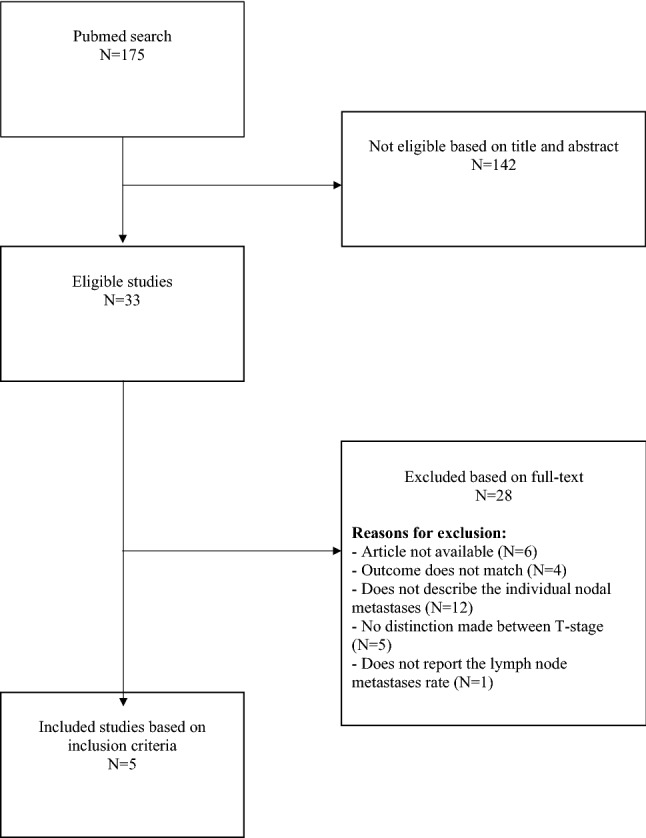
flowchart of the inclusion of articles (N)

The initial search resulted in 175 eligible articles. After screening on title and abstract 142 articles were excluded. The remaining 33 articles were retrieved for full-text evaluation. Five articles met the inclusion criteria. Figure 1 shows the flow chart for the inclusion.

### Characteristics of the included studies

An overview of the study characteristics is displayed in Table 2. The included studies were performed between 1989 and 2014. Three studies were performed retrospectively [19–21] and two studies prospectively [22, 23]. A total of 4864 patients were included, ranging from 328 to 1699 patients between studies. One study included solely proximal gastric tumors, one study included solely distal gastric tumors and three studies reported the prevalence of nodal metastases for the individual LN stations per T-stage of tumors distributed in the whole stomach; proximal, middle and distal gastric tumors. Three studies performed a LND with the extend of at least a D2 LND [20, 21, 23]. One study reviewed solely metastases in LN station number 1 to 11 [19]. One study performed a D1, D1 + or a D2 LND, in which the extent of the LND was determined according to the characteristics of the tumor [22]. Four studies reported the prevalence of nodal metastases for T1 gastric tumors [19–22], three studies for T2 gastric tumors [20, 22, 23], two studies for T3 gastric tumors [21, 23] and three studies for T4 gastric tumors [20, 22, 23].

**Table 2:** Study characteristics

Study	Year	Retro-/prospective	Patients	Tumor location	Extent of lymphadenectomy	T-stage
Song et al. [20]	2014	Retrospective	328	prox	D2 or D2 +	T1-T4
Han et al. [19]	2011	Retrospective	650	Prox, mid, dist	1st and 2nd tier lymph nodes	T1
Kong et al. [22]	2011	Prospective	1050	distal	D1, D1 + or D2	T1-T4
Maruyama et al. [23]	1989	Prospective	1699	Prox, mid, dist	D2	T2, T4
Namieno et al. [21]	1996	Retrospective	1137	Prox, mid, dist	D2	T1

### Methodological quality

The quality of the articles assessed with the use of the CASP score system ranged between 5 and 10 points. One article was assessed as “low quality” [19], one articles was assessed as “moderate quality” [20] and three articles were assessed as “high quality” [21–23]. The methodological quality is displayed in Table 3.

**Table 3:** Critical appraisal

Study	A) Are the results of the study valid?							B) What are the results			C) Will the results help locally			Total score	Article quality
	Question 1	Question 2		Question 3	Question 4	Question 5	Question 6	Question 7	Question 8	Question 9	Question 10	Question 11	Question 12		
	Did the study address a clearly focused issue?	Was the cohort recruited in an acceptable way?	Is it worth continuing?	Was the exposure accurately measured to minimise bias?	Was the outcome accurately measured to minimise bias?	a) Did the authors identified all important confounding factors? b) Have they taken account of the confounding factors in the design and/or analysis	a) Was the follow up of subjects complete enough? b) Was the follow up of subjects long enough?	What are the results of this study?	How precise are the results?	Do you believe the results?	Can the results be applied to the local population?	Do the results of this study fit with other available evidence?	What are the implications of this study for practice?		
	score	score		score	score	score	score	score	score	score	score	score	score		
Song et al	1	1	1	1	1	0	1	0	0	1	0	1	0	7	moderate
Han et al	1	1	1	0	0	0	1	0	0	1	0	1	0	5	low
Kong et al	1	1	1	1	1	0	1	1	1	1	0	1	1	10	high
Maruyama et al	1	1	1	1	1	1	1	0	1	1	0	1	1	10	high
Namieno et al	1	1	1	1	1	0	1	0	0	1	0	1	0	9	high

### Prevalence of lymph node metastases

An overview of the prevalence of LN metastases is shown in Table 4. The prevalence of nodal metastases in the individual LN stations for the different T-stage is shown separately for every article. An overall mean of the five studies is shown in Table 5.

**Table 4:** Overview of the lymph node metastases rate of the included studies (*n*/*N*(%))

LN station	T1	T2	T3	T4
*Song et al*				
1	1/15 (6.7%)	6/26 (23%)	78/177 (44.1%)	43/110 (39.1%)
2	1/15 (6.7%)	4/26 (15.4%)	54/177 (30.5%)	36/110 (32.7%)
3	2/15 (13.3%)	6/26 (23.1%)	70/177 (39.5%)	57/110 (51.8%)
4	0/15	1/26 (3.9%)	24/177 (13.9%)	23/110 (20.9%)
5	0/15	1/26 (3.9%)	12/177 (6.8%)	13/110 (11.8%)
6	0/15	1/26 (3.9%)	13/177 (7.3%)	14/110 (12.7%)
7	0/15	3/26 (11.5%)	40/177 (22.6%)	32/110 (29.1%)
8	0/15	2/26 (7.7%)	14/177 (7.9%)	17/110 (15.4%)
9	0/15	0/26	10/177 (5.6%)	7/110 (6.4%)
10	0/15	0/26	16/177 (9%)	13/110 (11.8%)
11	0/15	2/26 (7%)	10/177 (5.6%)	15/110 (13.6)
12	0/15	1/26 (3.9%)	7/177 (4%)	9/110 (8.2%)
13			1/177 (0.6%)	3/110 (2.7%)
14			3/177 (1.7%)	4/110 (3.6%)
15			0/177	2/110 (1.8%)
16			4/177 (2.3%)	11/110 (10.0%)
19		0/26	1/177 (0.6%)	1/110 (0.9%
20		0/26	0/177	1/110 (0.9%
Total	2/15 (13.33%)	12/26 (46.2%)	129/177 (72.9%)	88/110 (80%)
*Han et al*				
1	6/650 (0.9%)			
2	1/650 (0.2%)			
3	44/650 (6.8%)			
4	23/650 (3.5%)			
5	1/650 (0.2%)			
6	18/650 (2.8%)			
7	4/650 (0.6%)			
8	3/650 (0.5%)			
9	4/650 (0.6%)			
10	0/650			
11	1/650 (0.2%)			
	64/650 (9.8%)			
*Kong et al*				
1	3/643 (0.5%)	9/131 (6.9%)	10/175 (5.7%)	6/92 (6.5%)
2	0/20	0/1	0/10	0/13
3	29/650 (4.5%)	18/131 (13.7%)	78/176 (44.3%)	61/93 (65.6%)
4	19/650 (2.9%)	16/131 (12.2%)	46/176 (26.1%)	54/93 (58.1%)
5	8/627 (1.3%)	11/124 (8.9%)	25/172 (14.5%)	27/88 (30.7%)
6	30/650 (4.6%)	32/131 (24.4%)	65/176 (36.9%)	54/92 (58.7%)
7	16/649 (2.5%)	14/129 (10.9%)	36/176 (20.5%)	29/93 (31.2%)
8	10/637 (1.6%)	13/129 (10.1%)	35/172 (20.3%)	27/92 (29.3%)
9	0/601	1/126 (0.8%)	6/165 (3.6%)	9/86 (10.5%)
10	0/15	0/3	1/11 (9.1%)	1/8 (12.5%)
11	4/572 (0.7%)	3/120 (2.5%)	7/158 (4.4%)	15/86 (17.4)
12	3/425 (0.7%)	7/109 (6.4%)	7/149 (4.7%)	10/76 (13.2)
14	1/271 (0.4%)	3/101 (3.0%)	4/133 (3.0%)	8/76 (10.5%)
*Namieno et al*				
1	7/1137 (0.6%)			
2	1/1137 (0.1%)			
3	60/1137 (5.3%)			
4	37/1137 (3.3%)			
5	4/1137 (0.4%)			
6	24/1137 (2.1%)			
7	12/1137 (1.1%)			
8	6/1137 (0.5%)			
9	7/1137 (0.6%)			
14	1/1137 (0.1%)			
Total	108/1137 (9.5%)			
*Maruyama et al*				
1		16/328 (4.9%)		326/1371 (23.8%)
2		7/328 (2.1%)		233/1371 (17.0%)
3		82/328 (25.0%)		1000/1371 (72.9%)
4		52/328 (15.9%)		609/1371 (44.4%)
5		7/328 (2.1%)		115/1371 (8.4%)
6		56/328 (17.1%)		472/1371 (34.4%)
7		30/328 (9.1%)		504/1371 (36.8%)
8		23/328 (7.0%)		399/1371 (29.1%)
9		16/328 (4.9%)		280/1371 (20.4%)
10		3/328 (0.9%)		120/1371 (8.8%)
11		7/328 (2.1%)		193/1371 (14.1%)
12		7/328 (2.1%)		115/1371 (8.4%)
13		0/328		37/1371 (2.7%)
14		0/328		55/1371 (4.0%)
15		0/328		9/1371 (0.7%)
16		0/328		115/1371 (8.4%)

**Table 5:** Overall lymph node metastasis rate of the five included studies (*n*/*N*(%))

LN station	T1	T2	T3	T4
1	17/2445 (0.7%)	31/485 (6.4%)	88/352 (25.0%)	375/1573 (23.8%)
2	3/1822 (0.2%)	11/355 (3.1%)	54/187 (28.9%)	269/1494 (18.0%)
3	135/2452 (5.5%)	106/485 (21.9%)	148/353 (41.9%)	1118/1574 (71,0%)
4	79/2452 (3.2%)	69/485 (14.2%)	70/353 (19.8%)	686/1574 (43.6%)
5	13/2429 (0.5%)	19/478 (4.0%)	37/349 (10.6%)	155/1569 (9.9%)
6	72/2452 (2.9%)	89/485 (18.4%)	78/353 (22.1%)	540/1573 (34.3%)
7	32/2451 (1.3%)	47/483 (9.7%)	76/353 (21.5%)	565/1574 (35.9%)
8	19/2439 (0.8%)	38/483 (7.9%)	49/349 (14.0%)	443/1573 (28.2%)
9	11/2403 (0.5%)	17/480 (3.5%)	16/342 (4.7%)	296/1567 (18.9%)
10	0/680	3/357 (0.8%)	17/188 (9.0%)	134/1489 (9.0%)
11	5/1237 (0.4%)	12/474 (2.5%)	17/335 (5.1%)	223/1567 (14.2%)
12	3/440 (0.7%)	15/463 (3.2%)	14/326 (4.3%)	134/1557 (8.6%)
13		0/354	1/177 (0.6%)	40/1481 (2.7%)
14	2/1408 (0.1%)	3/455 (0.7%)	7/310 (2.3%)	67/1557 (4.3%)
15		0/354	0/177	11/1481 (0.7%)
16		0/354	4/177 (2.3%)	126/1481 (8.5%)
19		0/26	1/177 (0.6%)	1/110 (0.9%)
20		0/26	0/177	1/110 (0.9%)

#### Lymph node metastases rate in tumors limited to mucosa or submucosa (T1)

**Fig. 2:**
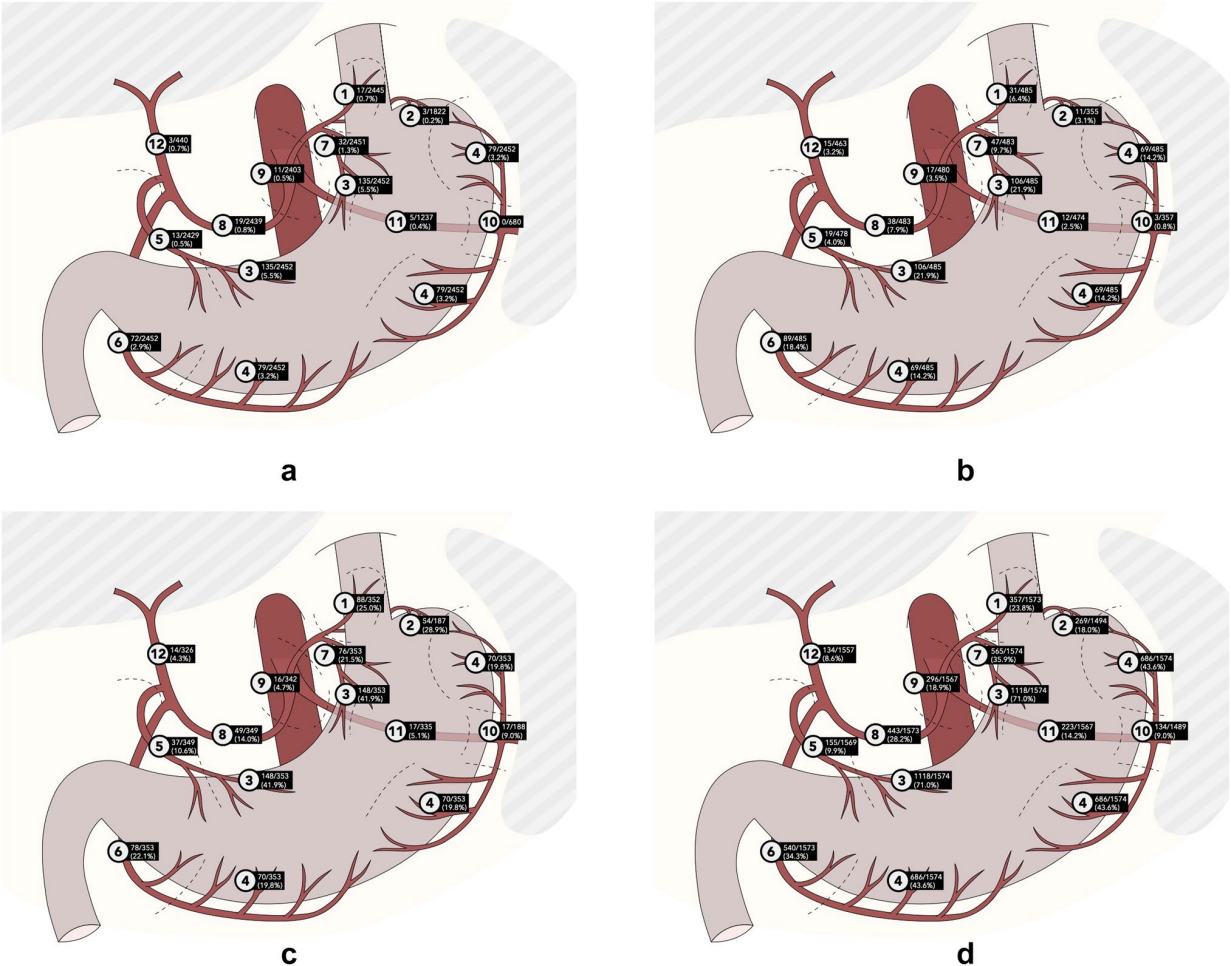
**a** Overall lymph node metastases rates in T1 gastric tumors, station 1–12. **b** Overall lymph node metastases rates in T2 gastric tumors, station 1–12. **c** Overall lymph node metastases rates in T3 gastric tumors, station 1–12. **d** Overall lymph node metastases rates in T4 gastric tumors, station 1–12

For T1 GC, the analysis included a total of 2452 patients, reported by four articles. If LN metastases were found in the presence of a T1 gastric carcinoma, the LNs along the lesser gastric curvature (station 3) show the highest metastases rate (5.5%). Concerning the LN stations 8 to 20, the LNs around the common hepatic artery (station 8) show the highest metastases rate (0.8%). There were no studies reporting the metastases rate for the LN stations 13, 15, 16, 19, 20. There were no positive LN metastases reported in LN station 10. An illustration of the LN stations 1–12, with regard to the metastases rate is shown in Fig. 2a.

#### Lymph node metastases rate in tumors with muscularis propria involvement, without penetrating the serosa (T2)

For T2 GC, the analysis included a total of 485 patients, reported by three articles. If LN metastases were found in the presence of a T2 gastric carcinoma, the LNs along the lesser gastric curvature (station 3) show the highest metastases rate (21.9%). Concerning the LN stations 8 to 20, the LNs around the common hepatic artery (station 8) show the highest metastases rate (3.2%). There were no studies reporting the metastases rate for the LN stations 13, 15, 16. There were no positive LN metastases reported in the LN stations 19 and 20. An illustration of the LN stations 1–12, with regard to the metastases rate is shown in Fig. 2b.

#### Lymph node metastases rate in tumors with subserosal involvement, without penetrating adjacent structures (T3)

For T3 GC, the analysis included a total of 353 patients, reported by two articles. If LN metastases were found in the presence of a T3 gastric carcinoma, the LNs along the lesser gastric curvature (station 3) show the highest metastases rate (41.9%). Concerning the LN stations 8 to 20, the LNs around the common hepatic artery (station 8) show the highest metastases rate (14.0%2). There were no positive LN metastases reported in LN station 15 and LN station 20. An illustration of the LN stations 1–12, with regard to the metastases rate is shown in Fig. 2c.

#### Lymph node metastases rate in tumors invading the serosa or adjacent structures (T4)

For T4 GC the analysis included a total of 1574 patients, reported by three articles. If LN metastases were found in the presence of a T4 gastric carcinoma, the LNs along the lesser gastric curvature (station 3) show the highest metastases rate (71.0%). Concerning the LN stations 8 to 20, the LNs around the common hepatic artery (station 8) show the highest metastases rate (28.2%). An illustration of the LN stations 1–12, with regard to the metastases rate is shown in Fig. 2d.

## Discussion

This systematic review investigated the prevalence of nodal metastases for the individual LN stations between different T-stages. The results show a correlation of the prevalence of nodal metastases between the different LN stations and different T-stages, with an overall low prevalence in early, T1 gastric carcinomas and an overall high prevalence in the more advanced, T3 and T4 gastric carcinomas.

No previous systematic review reported the prevalence of nodal metastases in the individual LN stations. To determine the adequate extent of LND, it is critical to understand which LN stations are generally affected for a specific gastric T-stage. In addition, this is crucial to determine whether a D1 LND is justified for early, T1-stage GC and whether a D2 LND is justified for more advanced gastric carcinomas.

This review is consistent with previous literature studies reporting the metastases rate for the different T-stages [12, 24, 25]. These studies similarly show an overall low metastases rate in the early carcinomas and an overall high prevalence in the more advanced carcinomas. However, these studies do not report the prevalence of nodal metastases in the individual LN stations.

There are limited studies reporting the prevalence of nodal metastases for the individual LNs per T-stage. As a result, only five articles could be included. There was a considerable heterogeneity between studies; not every article reported the metastases rate in every individual LN station for every T-stage. No recently performed studies were eligible for inclusion. The 7th rather than the 8th edition of the AJCC Cancer Staging Manual was used because all included articles were performed between 1989 and 2014.

In addition to gastric T-stage, tumor location plays a significant role in the LN metastases pattern and extent of LND [26]. Furthermore, histological and immunohistochemical characteristics and molecular profiling affect the prognosis [27, 28]. As a result of the considerable heterogeneity between studies; including considerable differences in distribution of tumor location, differences in histology and molecular profiling could not be processed in this systematic review. The combination of T-stage and tumor location could, however, provide a further understanding of the extent of LND for a more personalized approach.

All included studies were conducted in Asia. The results of this study endorse a more tailored approach based on gastric T-stage. However, these results cannot directly be extrapolated to a Western population as tumor biology and neoadjuvant chemotherapy regimens differ. Studies that investigate the distribution of LN metastases in a Western GC population are needed.

While preoperative staging is essential for deciding on the treatment strategy, the accuracy of GC staging is known to be limited. It remains unclear which preoperative imaging modality for GC should be regarded as optimal [29]. Current multidetector computerized tomography in combination with endoscopic ultrasonography has increased the accuracy of clinical gastric tumor staging [30]. However, detecting peritoneal and nodal metastases remains uncertain [31, 32]. Using a multimodality strategy, accuracy for clinical lymph node staging ranges between 50 and 90% [33]. Even with an increased accuracy of clinical tumor staging, a significant deviation between the clinical and the pathological T-stage remains [34]. With important consequences for the clinical applicability.

Neoadjuvant chemotherapy is considered a standard treatment for locally advanced gastric cancer. While still debated in some Eastern countries [35, 36], neoadjuvant chemotherapy has been widely used in the treatment of patients with locally advanced GC [37, 38]. However, neoadjuvant chemotherapy influences the tumor and potentially downstages the N-stage and T-stage. As a result, the deviation between clinical and pathological T-stage will increase. Previous studies showed a considerable downstaging of tumor stage after neoadjuvant chemotherapy together with poor accuracy of preoperative staging [39, 40]. The included studies in this review do not report whether neoadjuvant chemotherapy was administrated. Studies focusing on the optimalisation of imaging modalities in GC are awaited.

This systematic review showed an overall high prevalence of nodal metastases in the more advanced gastric carcinoma suggesting that a D2 LND should be considered for T2-4 tumors. Furthermore, it showed an overall low prevalence of nodal metastases in the early gastric carcinomas, especially in the individual LN station 8–12, suggesting that possibly a D1 LND could be adequate for T1 tumors who are not suitable for endoscopic resection.

## Conclusion

This systematic review provides an overview of the prevalence of nodal metastases for the individual LN stations between different T-stages. No significant recommendations could be drawn from this analysis due to the limited evidence. However, an overall low prevalence of nodal metastases in the individual LN stations in early T1 gastric carcinomas and an overall high prevalence in more advanced T3 and T4 gastric carcinomas endorse a more tailored approach based on the different gastric T-stages. In addition, a less extensive LND seems justified in early T1 carcinoma.

## Abbreviations

GC: Gastric cancer
LN: Lymph node
LND: Lymph node dissection/lymphadenectomy

## References

[1] Ferlay J, Soerjomataram I, Dikshit R, Eser S, Mathers C, Rebelo M (2015). Cancer incidence and mortality worldwide: sources, methods and major patterns in GLOBOCAN 2012. Int J Cancer.

[2] Wang W, Li YF, Sun XW, Chen YB, Li W, Xu DZ (2010). Prognosis of 980 patients with gastric cancer after surgical resection. Chin J Cancer.

[3] Marrelli D, De Stefano A, de Manzoni G, Morgagni P, Di Leo A, Roviello F (2005). Prediction of recurrence after radical surgery for gastric cancer: a scoring system obtained from a prospective multicenter study. Ann Surg.

[4] Shin C-H, Lee W-Y, Hong S-W, Chang Y-G (2016) Characteristics of gastric cancer recurrence five or more years after curative gastrectomy. Chin J Cancer Res 10.21147/j.issn.1000-9604.2016.05.05 10.21147/j.issn.1000-9604.2016.05.05 PMC5101224 27877009

[5] Barchi LC, Yagi OK, Jacob CE, Mucerino DR, Ribeiro U, Marrelli D (2016). Predicting recurrence after curative resection for gastric cancer: external validation of the Italian Research Group for Gastric Cancer (GIRCG) prognostic scoring system. Eur J Surg Oncol.

[6] Japanese Gastric Cancer Association (2021). Japanese gastric cancer treatment guidelines 2018 (5th edition). Gastric Cancer.

[7] Schmidt B, Yoon SS (2013). D1 versus D2 lymphadenectomy for gastric cancer. J Surg Oncol.

[8] Degiuli M, Sasako M, Ponti A (2010). Morbidity and mortality in the Italian Gastric Cancer Study Group randomized clinical trial of D1 versus D2 resection for gastric cancer. Br J Surg.

[9] Cuschieri A, Weeden S, Fielding J, Bancewicz J, Craven J, Joypaul V (1999). Patient survival after D1 and D2 resections for gastric cancer: long-term results of the MRC randomized surgical trial. Surgical Co-operative Group. Br J Cancer.

[10] Bonenkamp JJ, Hermans J, Sasako M, van de Velde CJ, Welvaart K, Songun I, et al (1999) Dutch Gastric Cancer Group. Extended lymph-node dissection for gastric cancer. N Engl J Med 10.1056/NEJM1999032534012 10.1056/NEJM199903253401202 10089184

[11] Smyth EC, Verheij M, Allum W, Cunningham D, Cervantes A, Arnold D (2016). Gastric cancer: ESMO clinical practice guidelines for diagnosis, treatment and follow-up. Ann Oncol Off J Eur Soc Med Oncol.

[12] Abdelfatah MM, Barakat M, Lee H, Kim JJ, Uedo N, Grimm I (2018). The incidence of lymph node metastasis in early gastric cancer according to the expanded criteria in comparison with the absolute criteria of the Japanese Gastric Cancer Association: a systematic review of the literature and meta-analysis. Gastrointest Endosc.

[13] Choi AH, Nelson RA, Merchant SJ, Kim JY, Chao J, Kim J (2016). Rates of lymph node metastasis and survival in T1a gastric adenocarcinoma in western populations. Gastrointest Endosc.

[14] Bravo Neto GP, dos Santos EG, Victer FC, Carvalho CE (2014). Lymph node metastasis in early gastric cancer. Rev Col Bras Cir.

[15] Liu X, Long Z, Cai H, Huang H, Shi Y, Wang Y (2014). Analysis of lymph node metastasis correlation with prognosis in patients with T2 gastric cancer. PLoS ONE.

[16] Page M J, McKenzie J E, Bossuyt P M, Boutron I, Hoffmann T C, Mulrow C D et al (2021) The PRISMA 2020 statement: an updated guideline for reporting systematic reviews BMJ 10.1371/journal.pmed.1003583 10.1186/s13643-021-01626-4 PMC8008539 33781348

[17] Washington K (2010). 7th edition of the AJCC cancer staging manual: stomach. Ann Surg Oncol.

[18] Association JGC (1998) Japanese Classification of Gastric Carcinoma - 2nd English Edition. Gastric Cancer 10.1007/s101209800016 10.1007/s101209800016 11957040

[19] Han KB, Jang YJ, Kim JH, Park SS, Park SH, Kim SJ (2011). Clinical significance of the pattern of lymph node metastasis depending on the location of gastric cancer. J Gastric Cancer.

[20] Song W, Liu Y, Ye J, Peng J, He W, Chen J, et al (2014) Proximal gastric cancer: lymph node metastatic patterns according to different T stages dictate surgical approach. Chin Med J (Engl) 25430447

[21] Namieno T, Koito K, Higashi T, Sato N, Uchino J (1996). General pattern of lymph node metastasis in early gastric carcinoma. World J Surg.

[22] Kong SH, Yoo MW, Kim JW, Lee HJ, Kim WH, Lee KU (2011). Validation of limited lymphadenectomy for lower-third gastric cancer based on depth of tumour invasion. Br J Surg.

[23] Maruyama K, Gunven P, Okabayashi K, Sasako M, Kinoshita T (1989). Lymph node metastases of gastric cancer. General pattern in 1931 patients. Ann Surg.

[24] Hatta W, Gotoda T, Kanno T, Yuan Y, Koike T, Moayyedi P (2020). Prevalence and risk factors for lymph node metastasis after noncurative endoscopic resection for early gastric cancer: a systematic review and meta-analysis. J Gastroenterol.

[25] Jiang B, Zhou L, Lu J, Wang Y, Guo J (2020). Predictors of lymph node metastasis and residual tumor in early gastric cancer patients after noncurative endoscopic resection: a systematic review and meta-analysis. Ther Adv Gastroenterol.

[26] Akagi T, Shiraishi N, Kitano S (2011). Lymph node metastasis of gastric cancer. Cancers.

[27] Hu B, El Hajj N, Sittler S, Lammert N, Barnes R, Meloni-Ehrig A (2012). Gastric cancer: classification, histology and application of molecular pathology. J Gastrointest Oncol.

[28] Gurzu S, Orlowska J, Sugimura H, Bara T, Szentirmay Z, Januszewicz et al (2017) Immunohistochemical features and staging of early gastric cancer. Arch Med Sci 10.5114/aoms.2016.58665 10.5114/aoms.2016.58665 PMC5701676 29181068

[29] Nie RC, Yuan SQ, Chen XJ, Chen S, Xu LP, Chen YM (2017). Endoscopic ultrasonography compared with multidetector computed tomography for the preoperative staging of gastric cancer: a meta-analysis. World J Surg Oncol.

[30] Giganti F, Orsenigo E, Arcidiacono PG, Nicoletti R, Albarello L, Ambrosi A (2016). Preoperative locoregional staging of gastric cancer: is there a place for magnetic resonance imaging? Prospective comparison with EUS and multidetector computed tomography. Gastric Cancer.

[31] Lee MH, Choi D, Park MJ, Lee MW (2012). Gastric cancer: imaging and staging with MDCT based on the 7th AJCC guidelines. Abdom Imaging.

[32] Lee IJ, Lee JM, Kim SH, Shin CI, Lee JY, Kim SH (2010). Diagnostic performance of 64-channel multidetector CT in the evaluation of gastric cancer: differentiation of mucosal cancer (T1a) from submucosal involvement (T1b and T2). Radiology.

[33] Vergadis C, Schizas D (2018). Is accurate N - staging for gastric cancer possible?. Front Surg.

[34] Fairweather M, Jajoo K, Sainani N, Bertagnolli MM, Wang J (2015). Accuracy of EUS and CT imaging in preoperative gastric cancer staging. J Surg Oncol.

[35] Mihmanli M, Ilhan E, Idiz UO, Alemdar A, Demir U (2016). Recent developments and innovations in gastric cancer. World J Gastroenterol.

[36] Ganschow P, Hofmann L, Stintzing S, Heinemann V, Angele M, Werner J (2021). Operative results and perioperative morbidity after intensified neoadjuvant chemotherapy with FLOT for gastroesophageal adenocarcinoma impact of intensified neoadjuvant treatment. J Gastrointest Surg.

[37] National Health Commission of the People’s Republic of China (2019) Chinese guidelines for diagnosis and treatment of gastric cancer 2018 (English version). Chin J Cancer Res 10.21147/j.issn.1000-9604.2019.05.01 PMC6856703 31814675

[38] Ajani JA, D’Amico TA, Almhanna K, Bentrem DJ, Chao J, Das P (2016). Gastric cancer, version 3.2016, NCCN Clinical Practice guidelines in oncology. J Natl Compr Canc Netw.

[39] Prasad P, Sivaharan A, Navidi M, Fergie BH, Griffin SM, Phillips AW (2022). Significance of neoadjuvant downstaging in gastric adenocarcinoma. Surgery.

[40] Levenson G, Voron T, Paye F, Balladur P, Debove C, Chafai N (2021). Tumor downstaging after neoadjuvant chemotherapy determines survival after surgery for gastric adenocarcinoma. Surgery.

